# Pharmacological interventions for the management of anesthesia and sedation in patients with Duchenne muscular dystrophy: a systematic review and meta-analysis

**DOI:** 10.3389/fmed.2025.1497538

**Published:** 2025-01-27

**Authors:** Xianghong Lian, Yang Jing, Ting Luo, Yixin Guo, Yunzhu Lin

**Affiliations:** ^1^Department of Pharmacy, West China Second University Hospital, Sichuan University, Chengdu, China; ^2^Evidence-Based Pharmacy Center, West China Second University Hospital, Sichuan University, Chengdu, China; ^3^Key Laboratory of Birth Defects and Related Diseases of Women and Children, Sichuan University, Ministry of Education, Chengdu, China

**Keywords:** Duchenne muscular dystrophy, sedative, anesthesia, pharmacological interventions, systematic review, meta-analysis

## Abstract

**Background:**

Patients with duchenne muscular dystrophy (DMD) have an increased risk of complications when they undergo sedation or general anesthesia. However, due to improvements in cardiopulmonary therapies during anesthetic care, patients with DMD are experiencing an unprecedented duration of survival. We performed a systematic analysis to assess the benefits and risks of pharmacological interventions for the management of anesthesia and sedation in DMD patients.

**Methods:**

We included any type of study reporting any drug intervention to manage anesthesia and sedation in participants previously diagnosed with DMD. Our primary outcomes were the onset time, recovery time, and neurodevelopmental disabilities. Seven electronic databases and three clinical trial registry platforms were searched. Data from the eligible studies were combined to calculate pooled risk ratios or standardized mean differences, and some included studies are presented in a narrative synthesis.

**Results:**

Forty studies with 196 DMD participants were included in the analysis. Compared with those of the control group, the sensitivity of patients with DMD to neuromuscular blocking agents (NMBAs) may have resulted in a prolonged onset time [MD = −0.96, 95% CI (0.71, 2.60), *I*^2^ = 33%, *P* < 0.0001] and recovery time [MD = 2.22, 95% CI (1.14, 3.30), *I*^2^ = 76%, *P* < 0.0001] from anesthesia. The neuromuscular blocking effects showed a significant age dependence in DMD patients, and the safe use of 2 mg/kg sugammadex to antagonize deep neuromuscular blockade and rapid recovery has been reported. Furthermore, DMD patients are at risk of developing malignant hyperpyrexia with general/inhaled anesthesia, and dantrolene is often used for effective rescue. In addition, general anesthesia and central neuraxial blockade in patients with severe DMD are unsafe because respiratory depression and myocardial complications may occur after the administration of volatile anesthetics and depolarizing muscle relaxants (succinylcholine) during the induction of anesthesia.

**Conclusions:**

Patients with DMD are more sensitive to NMBAs with delayed onset times and prolonged recovery times. Precautions for DMD patients should include quantitative neuromuscular monitoring, electrocardiographic monitoring and rapid airway protection throughout anesthesia. Compared with general anesthesia, regional anesthesia may be a relatively safe option.

## 1 Introduction

Duchenne muscular dystrophy (DMD) is a progressive neuromuscular disease transmitted by X-linked inheritance with an incidence of ~1 in 3,500 live male births (1, 2). The onset of clinical symptoms usually occurs during early childhood, and progression of the disease leads to a loss of ambulation in late childhood. At present, no cure exists for DMD, and treatment is aimed at minimizing symptoms (3).

Since patients with DMD present a wide range of symptoms, the associated treatment of health concerns is particularly necessary for each individual patient. Thus, patients with DMD often require anesthetic care during muscle biopsy or correction of progressive orthopedic deformities (4). Depending on the type of surgical procedure and the neurocognitive level of the patient, options include general anesthesia, regional anesthesia or procedural sedation (5, 6). However, the potential impacts of DMD on perioperative morbidity and even mortality cannot be ignored, as the literature has suggested a significantly increased risk during anesthetic care in these patients (8, 9). Earlier reports have outlined the potential for perioperative mortality with cardiac arrest and death in 2 of 25 patients requiring anesthetic care (10).

However, more recent reports have shown that with a better understanding of the pathophysiology of the disease, end-organ involvement and improvements in favorable perioperative care outcomes are possible even in this challenging patient population. In a review of 91 DMD patients who underwent 232 orthopedic surgical procedures, Muenster et al. reported no severe anesthesia-related complications and no cases of unexplained fever or rhabdomyolysis (11). Furthermore, in nearly all patients, neuromuscular blockade agents (NMBAs) were used; therefore, the complete spontaneous recovery of neuromuscular blockade (NMB) in DMD patients remains unclear, and the safest anesthetic technique has yet to be established (12–16).

Given the indispensable nature of sedatives and/or analgesia in DMD surgery/diagnosis and the frequency with which medically compromised DMD patients present for treatment, an increased need exists for reliable data that can inform clinical decision-making. Moreover, the relevant question of whether developments and changes in anesthetic techniques in recent years have improved the safety of anesthesia in this special group of DMD patients has gradually attracted widespread attention, but no studies have been published. Thus, the present review aimed to search for current evidence related to the use of analgosedation in the context of both the drugs used and their side effects and to formulate recommendations in this respect. This review is an up-to-date summary of the medical literature concerning this topic and identifies areas in need of future research.

## 2 Materials and methods

This systematic review and meta-analysis was performed according to the recommendations in the Preferred Reporting Items for Systematic Reviews and Meta Analyses (PRISMA) statement and the guidelines described in the Cochrane Handbook (17).

### 2.1 Search strategy

Our search comprised three English electronic databases (PubMed, Embase, and Cochrane Library) and four Chinese electronic databases (China National Knowledge Infrastructure, Wan Fang Database, Chinese Biomedical Literature Database, and VIP Database for Chinese Technical Periodicals). Three clinical trial registry platforms were used to identify additional studies, including Clinical Trials.gov, the World Health Organization Clinical Trials Registry Platform and the Cochrane Central Registry of Controlled Trials. The search strategy was specific for each database and included a combination of medical subject headings and free text terms (“DMD” or “Duchenne muscular dystrophy”) and (“sedation” or “anesthesia” or “analgosedation”). We looked for additional studies in the reference lists of the selected articles and contacted the authors when the information was unclear. The deadline for the retrieval of all studies was September 2024.

### 2.2 Inclusion criteria

The following studies were included: (1) studies examining human participants of any age and sex who were previously diagnosed with DMD; (2) any type of drug intervention used for the management of pain and/or sedation; (3) type of study—randomized/non-randomized controlled trials (RCTs), observational studies, case series, and case reports reporting on patients with previously diagnosed DMD; (4) outcomes—the degree and effectiveness of sedation provided by different pharmacological agents, feasibility, and tolerability were assessed, whereas the secondary outcomes included other adverse events. The exclusion criteria were as follows: (1) studies with incomplete or missing information; (2) studies were not published in Chinese or English; (3) abstracts from conferences and unpublished data; (4) no outcomes related to sedative/narcotic drugs or a lack of a specific sedative/narcotic drug detailed regimen.

### 2.3 Data extraction

Two authors independently extracted the data using a previously designed data extraction table. The data extracted were the authors, year of publication, country, experimental design, sample size, mean age, intervention measure, dose, type of procedure, and any outcome that met the inclusion criteria.

Two independent reviewers screened all the titles and abstracts to identify potentially eligible articles. They independently applied the eligibility criteria to perform the final selection. When discrepancies occurred between the two reviewers regarding the inclusion of the articles, they discussed and identified the reasons to either include or exclude the articles and then made the final decision. If they could not reach an agreement, the final decision was made by a third reviewer.

### 2.4 Risk of bias assessment

The Interventions' (MINORS) tool was used to assess the risk of bias in non-randomized studies (18). The quality of case report studies was evaluated using the Joanna Briggs Institute of Australia (JBI) quality assessment tool (19).

### 2.5 Statistical analysis

The meta-analysis was conducted with RevMan 5.3. The data were pooled and reported as relative risks (RR) or Mean Difference (MD) with 95% confidence interval (CI). Heterogeneity was assessed using *I*-squared (*I*^2^) statistics. A fixed effects model was initially constructed. If significant heterogeneity existed among trials (*I*^2^ > 50%), potential sources of heterogeneity were considered, and where appropriate, a random effects model was used (20, 21). We planned to report outcome data in tables if a meta-analysis was deemed inappropriate, for example, because of clinical or statistical heterogeneity.

## 3 Results

### 3.1 Study search and characteristics

**Figure 1:**
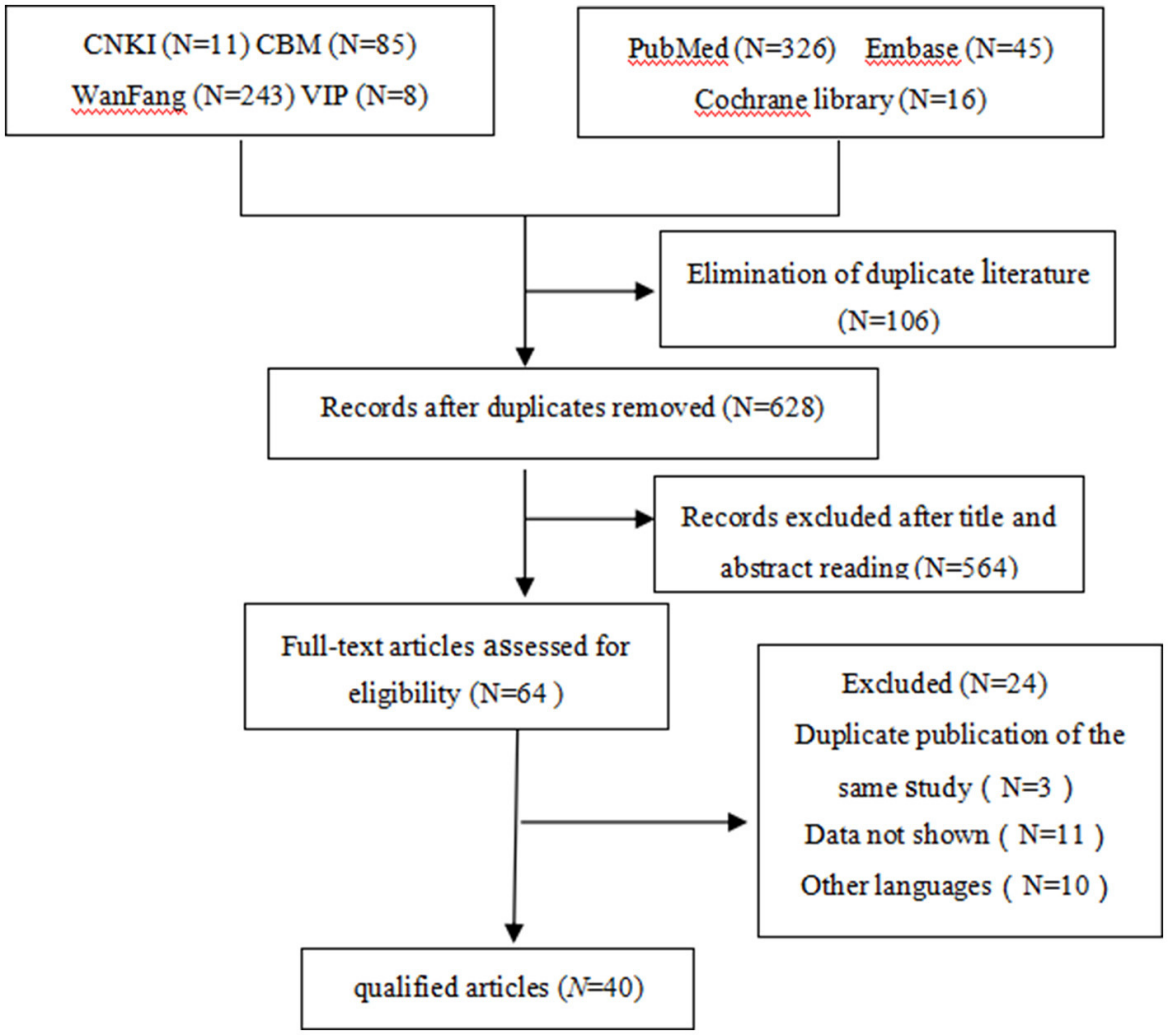
Flow diagram of selecting study.

The titles and abstracts of a total of 734 studies were screened, of which 670 were deemed irrelevant (Figure 1). The full texts of the remaining 64 studies were read, and 24 were excluded, leaving 40 studies with 196 patients to be included (35 case reports and five non-randomized controlled trials; Tables 1–3) (4, 7, 12–16, 22–54).

**Table 1:** Characteristics of included non-randomized controlled trials.

**References**	**Country**	**Sample size**	**Age (year)**	**Weight (kg)**	**Group**	**Main modeof anesthesia**	**Preoperativeevaluation**	**Anesthesia premedication**	**Anesthesiainduction**	**Anesthesiamaintenance**	**Operationduration**	**Postoperative analgesia**	**Side effects**	**Onset time (min)**	**Recovery time (min)**
Ihmsen et al. (50)	Iran	8	6.3 ± 1.6	25 ± 8	Control group children	General anesthesia	The plasma cholinesterase activity was within the normal range	Midazolam orally 45 min	2 g/kg fentanyl and 3 mg/kg propofol	8 mg/kg propofol and remifentanil	NA	NA	NA	2.0 (1.3–3.0)	8.5 (6.5–12.0)
		8	13.5 ± 2.6	54 ± 9							Control group adolescents	NA	NA	2.5 (2.0–3.5)	9.1 (7.5–9.8)
		11	7.9 ± 1.2	27 ± 6							DMD group children	NA	NA	2.2 (1.5–4.7)	12 (3.0–21)
		11	13.8 ± 1.5	54 ± 20							DMD group adolescents	NA	NA	4.0 (1.8–7.0)	18 (4.5–45)
Wick et al. (51)	Germany	12	13.3 ± 2.0	49.8 ± 13.6	DMD group	General anesthesia	ASA III	3.75 mg midazolam	2–3 μg/kg fentanyl, 3 mg/kg propofol	8–12 mg/kg propofol, remifentanil	5–7 h	NA	NA	203 (90–420) s	71.0 (39–144)
		12	13.8 ± 3.0	54.5 ± 15.9	Control group		ASA I				2–3 h	NA	NA	90 (60–195) s	16.8 (13–36)
Muenster et al. (52)	Germany	12	13.5 ± 1.7	60.5 ± 9.8	DMD Group	Generalanesthesia	Early left ventricular hypertrophy	3.75 mg midazolam orally 45 min	2–3 μg/kg fentanyl and propofol 3 mg/kg	8–12 mg/kg propofol, remifentanil	5–7 h	NA	NA	315 (120–465) s	72.0 (36–141)
		12	13.7 ± 2.8	53.8 ± 17.8	Control group		NA				2–3 h			195 (75–270) s	13.8 (7–18)
Ririe et al. (53)	USA	8	12 (11–15)	NA	DMD Group	General anesthesia	ASA I	1 mg/kg oral midazolam	10 μg/kg glycopyrrolate,4 mg/kg thiopental, 5–10 μg/kg fentanyl	2–5 μg/kg/h fentanyl, 0.03 mg/kg midazolam	NA	NA	NA	28 (15–43)	36 (13–52)
		8	12 (8–18)	NA	Control group						NA	NA	20 (14–33)	6 (4–9)
Kako et al. (54)	USA	24	9.7 ± 1.4	33.3 ± 7.7	DMD with 1 μg/kg dexmedetomidine	Procedural sedation	ASA nil per os	0.5 mg/kg midazolam, topical lidocaine cream	Dexmedetomidine and 1 mg/kg ketamine	1 μg/kg dexmedetomidine	21 ± 5 min	15 mg/kg acetaminophen	Airway obstruction; vomiting	3.7 ± 2.3	174 ± 58
		29	8.8 ± 1.8	30.2 ± 10.8	DMD with 0.5 μg/kg dexmedetomidine				Dexmedetomidine and 1 mg/kg ketamine	0.5 μg/kg dexmedetomidine	22 ± 7 min		Vomiting	2.8 ± 1.6	146 ± 65

**Table 2:** Characteristics of included case reports of DMD patients.

**References**	**Country**	**Sample size**	**Age (year)**	**Weight (kg)**	**Study population**	**Main mode ofanesthesia**	**Anesthesia premedication**	**Anesthesiainduction**	**Anesthesiamaintenance**	**Anesthesiareversedation**	**Operation duration**	**Postoperative analgesia**	**Side effects**
Kim and Chun (22)	Korea	1	11	53	DMD with percutaneous nephrolithotomy	General anesthesia	Glycopyrrolate 0.2 mg was injected intramuscularly	5 mg/kg opentothal sodium and 5 mg omidazolam	250 mg/kg/min propofol and 0.3 mg/kg/min oremifentanil	2.0 mg/kg sugammadex (106 mg)	90 min	NA	No adverse event
Jung et al. (24)	Korea	1	6	19	DMD with muscle biopsy	General anesthetia	No premedication	Midazolam 1 mg, fentanyl 25 μg, Rocuronium bromide 6 mg	Propofol	4 mg pyridostigmine and 0.16 mg glycopyrrolate	45 min	NA	No complication
Bang et al. (26)	Korea	1	22	47	DMD with a left distal femur fracture and needed to undergo reduction and internal fixation	Peripheral nerve blocks	20 ml of 0.375% ropivacaine	NA	15 and 5 ml of 0.375% ropivacaine into the femoral nerve and the lateral femoral	NA	40 min	NA	NA
Büget et al. (27)	Turkey	1	17	NA	DMD with upper extremity amputation	Regional anesthesia	1% lidocaine	30 ml 0.5% bupivacaine	NA	2 h	NA	Fever of 39°C	
de Boer et al. (12)	Netherlands	1	9	46	DMD with a humerus fracture	General anesthesia	1,000 mg paracetamol	8–12 mg/kg propofol, 0.05–0.20 μg/kg/min remifentanil,1.0 mg/kg rocuronium	4.0 mg/kg sugammadex (184 mg)	35 min	NA	Uneventful	
Wefki Abdelgawwad Shousha et al. (13)	Italy	1	25	NA	DMD with open cholecystectomy	General anesthesia	Ciprofloxacin 2 gm; metronidazole 500 mg; ondansetron 4 mg	Propofol 150 mg, fentanyl 200 mcg, and rocuronium bromide 10 mg	Fentanyl in a total dose of 400 mcg (200–100–100), rocuronium bromide 5 mg repeated every 45 min	150 mg sugammadex	240 min	NA	NA
Obata et al. (28)	Japan	1	11	40 kg	DMD with strabismus	Inhalational induction	NA	Sevoflurane 4%, nitrous oxide 66%	Sevoflurane 1.5–3.0%, nitrous oxide 64%	1 mg/kg dantrolene sodium	51 min	25 mg diclofenac	Rhabdomyolysis
Obata et al. (28)	Japan	1	11	40 kg	DMD with strabismus	Inhalational induction	NA	Sevoflurane 4%, nitrous oxide 66%	Sevoflurane 1.5–3.0%, nitrous oxide 64%	1 mg/kg dantrolene sodium	51 min	25 mg diclofenac	Rhabdomyolysis
Richa et al. (30)	France	3	11/13/ 10	31/35/ 38 kg	DMD wtih posterior spinal surgery	General anesthesia	1 mg/kg of hydroxyzine	1 μg/kg/min remifentanil, propofol 3–5 mg/kg	0.1–0.4 μg/kg/min remifentanil, 3–9 mg/kg/h propofol	NA	345 ± 31 min	Morphine 4 μg/kg, paracetamol 15 mg/kg	No adverse event
Saldanha et al. (31)	Brazil	1	5	20	DMD with tumor excision and cervical emptying	General anesthesia	5 mg midazolam	0.5 μg/kg/min remifentanil, 6 μg/ml propofol	0.3 μg/kg/min Remifentanil and 3 μg/ml propofol	NA	180 min	0.2 mg/kg nalbuphine, 50 mg/kg dipirone	Without intercurrences
		1	24	NA	DMD with cholelithiasis		15 mg propofol, topic lidocaine	2.9 μg/ml propofol, 0.3 μg/kg/min remifentanil	NA	NA	40 min	NA	NA
Kocabas et al. (32)	Turkey	1	5	15	DMD with correction of Fallot's Tetralogy	General anesthesia	NA	6 mg/kg ketamine, 0.02 mg/kg atropine,0.05 mg/kg midazolam and 2 μg/kg fentanyl, Rocuronium 0.6 mg/kg	15 μg/kg fentanyl and 2–5 mg/kg/h ketamine	NA	240 min	15 mg/kg rectal paracetamol, 0.05 mg/kg morphine i.v	NA
Smelt (15)	Netherlands	1	16	60	DMD with scoliosis correction	General anesthesia	NA	1.0 mg alfentanil, 160 mg propofol, 30 mg rocuronium	Piritramide and desflurane?	1.0 mg adrenaline	40 min	NA	Ventricular fibrillation
Irwin and Henderson (16)	Hong Kong	1	14	NA	DMD with posterior spinal fusion	General anesthetic	Temazepam	Propofol and alfentanil supplemented with 65% nitrous oxide, atracurium	Alfentanil with 65% nitrous oxide	Adrenaline 0.5 mg, nitrous oxide	45 min	PCA morphine pump	Asystole
Rajmala et al. (33)	India	1	9	36	DMD with ophthalmic surgery	General + regional anesthesia	NA	2 mg/kg propofol, 0.05 mg/kg vecuronium,	3 mg/kg propofol oxygen (33%), nitrous oxide (67%), 0.25% bupivacaine	0.05 mg/kg neostigmine, 0.02 mg/kg atropine	NA	NA	Respiratory efforts
Vandepitte et al. (35)	America	1	27	41	DMD with pathologic fracture	Intercostal nerve blocks	NA	4 ml of ropivacaine 0.75% (T7–12)		NA	NA	Paracetamol 500 mg/6 h	NA
Molyneux (36)	UK	1	36 woman	60.3	DMD with a 37 weeks'pregnant woman	Spinal-epidural anesthesia	NA	A total of 2.5 ml of 0.5% hyperbaric bupivacaine with diamorphine		NA	NA	NA	No adverse event
Van Obbergh et al. (37)	Belgium	1	6	16	DMD with bilateral hip osteotomies	General anesthesia	NA	5 mg ketamine, 60 mg thiopentone, 10 mg atracurium,	0.5 μg/kg/min remifentanil	NA	NA	Paracetamol 15 mg/kg	No adverse event
Horikoshi et al. (39)	Japan	1	4	16	DMD with inguinal hernia	General anesthesia	NA	3 mg remimazolam, 100 μg fentanyl, 1.0 mg/kg/min remifentanil, 15 mg/h remimazolam, 10 mg rocuronium	15 mg/h remimazolam, 1.0 mg/kg/min remifentanil	40 mg sugammadex	NA	15 mg flurbiprofen axetil	No complications
Sethna and Rockoff (40)	America	1	5	NA	DMD with muscle biopsy	Inhalation anesthesia	NA	Nitrous oxide, oxygen and halothane by face mask	Dantrole (total dose 9 mg/kg)	NA	NA	Cardiac arrest	
Chalkiadis and Branch (41)	UK	1	8	30.2	DMD with left orchidopexy	Inhalation anesthesia	No premedication	5 mg/kg thiopentone,1.6 μg/kg fentanyl	50% nitrous oxide, oxygen, isoflurane 1.5%	Dantrolene 1 mg/kg	35 min	NA	Cardiac arrest
Shafy et al. (42)	America	1	36	61.1	DMD with right ischial pressure ulcer	Regional anesthesia	4 mg midazolam	Superficial anesthesia of the skin, subcutaneous tissue was achieved with 1% lidocaine, 3% chloroprocaine	NA	NA	1,000 mg acetaminophen	No adverse event	
Rathi et al. (43)	India	1	34	76	DMD with radical mastectomy	Regional anesthetic	2% lignocaine	0.375% ropivacaine, dexamethasone 4 mg	NA	1 h	1 mg paracetamol	No adverse event	
Kulshrestha et al. (45)	India	1	12	48	DMD with dentigerous cyst	Procedural sedation	0.2 mg glycopyrrolate, 1 μg/kg fentanyl	Dexmedetomidine was administered slowly with a loading dose of 1 μg/kg over 15 min followed by a continuous infusion at 0.5 μg/kg/h		NA	40 min	NA	NA
Raman et al. (46)	America	1	9	45	DMD with esophagogastroduodenoscopy (EGD)	Sedation	15 mg midazolam	1 μg/kg dexmedetomidine, 1 mg/kg ketamine		NA	15 min	NA	NA
Rozmiarek et al. (7)	America	1	21	43	DMD with bone marrow aspiration	Sedation	NA	1 μg/kg Dexmedetomidine, 20 mg Ketamine		NA	NA	NA	NA
Wang and Stanley (47)	America	1	2	15	DMD with silicon implant	General anesthesia	0.2 mg atropine	0.25%−2.75% halothane, 50% nitrous oxide	0.1 mg atropine, 40 mg succinylcholine	15 mg dantrolene	NA	NA	Malignant hyperthermia
		1	3	15	DMD with muscle biopsy	General anesthesia	NA	350 mg methohexitone, 50% nitrous oxide,42 μg fentanyl, 7 mg atracurium	Fentanyl, 50% nitrous oxide	40 mg dantrolene	NA	NA	NA
Kim et al. (48)	Korea	1	20	39	DMD with LVAD implantation	General anesthesia	NA	4 mcg/ml propofol, 3 ng/ml remifentanil,40 mg rocuronium	2–2.5 mcg/ml propofol, 1–2 ng/ml remifentanil, 2 mcg/kg/min Rocuronium	NA	NA	1,000 mcg fentanyl, 0.3 mg ramosetron	No complications
Frankowski et al. (4)	America	2	10-year-old/9-year-old	54 kg/ 20 kg	DMD with heel cord surgeries	General anesthesia	1.5–2 mg Midazolam	1–2 mg/kg sodium thiopental, 1.5–2.0 mg/kg lidocaine, 2–5 mg/kg fentanyl, 2–4 mg/kg propofol	Propofol and remifentanil infusions at rates of 45–150 μg/kg/min	NA	2.5–3 h	NA	Uneventful

**Table 3:** Characteristics of included case reports of BMD patients.

**References**	**Country**	**Sample size**	**Age (year)**	**Weight (kg)**	**Study population**	**Main modeof anesthesia**	**Anesthesia premedication**	**Anesthesiainduction**	**Anesthesiamaintenance**	**Anesthesiareversedation**	**Operation duration**	**Postoperative analgesia**	**Side effects**
Parish and Farzin (23)	Iran	1	43	80	BMD with orthopedic surgery	General anesthesia	200 mg hydrocortisone	2 mg midazolam, 250 μg remifentanil and 60 mg lidocaine 2%	75–100 μg/kg/min propofol and 0.05–2 μg/kg/min remifentanil	NA	90 min	NA	No adverse event
Zhou et al. (25)	China	1	56 woman	48	BMD with laparoscopic hysterectomy and bilateral adnexectomy	General anesthesia	NA	2 mg midazolam, 16 mg etomidate, 20 μg sufentanil	75–100 μg /kg/min propofol and 0.05–2 μg/kg/m remifentanil,2% sevoflurane	NA	90 min	5 mg sufentanil and 30 mg ketorolac	No adverse event
Iwata et al. (29)	Japan	1	58	75 kg	BMD with laparoscopic cholecystectomy	General+ regional anesthesia.	NA	4 μg/ml propofol and 0.2 mg fentanyl, and 0.25 μg/kg/min remifentanil	2.5–3 μg/ml propofol; 0.75% ropivacaine, 1% lidocaine 20 ml	NA	NA	Flurbiprofen axetil 50 mg	No adverse event
Shimauchi et al. (14)	Japan	1	54	54	BMD with cholelithiasis	General anesthesia	NA	3 μg/kg fentanyl and 0.6 mg/kg midazolam,0.4 mg/kg Rocuronium	2–4 mg/kg/h propofol, 0.05–0.3 μg/kg/min remifentanil	100 mg sugammadex (2 mg/kg)	92 min	Pethidine (30 mg)	No adverse event
Jain (34)	India	1	34	NA	BMD with fistulectomy under saddle block	General + regional anesthesia	NA	1.8 ml of 0.5% bupivacaine L3–L4; 1.5 mg midazolam; 30 mg propofol.		Propofol was stopped, nebulized salbutamol	NA	NA	Coughing
Peng and Wei (38)	China	1	2	15	BMD with inguinal hernia	General + regional anesthesia	NA	0.15 mg atropine, 0.5 mg midazolam, 45 mg propofol, 30 μg fentanyl, 1 mg cisatracurium besylate. 0.25% ropivacaine 6 ml	2–4 mg/kg/h propofol, 0.05–0.1 μg/kg/min remifentanil	0.2 mg neostigmine, 0.1 mg atropine	20 min	NA	No adverse event
Kawaai et al. (44)	Japan	1	19	59	BMD with dental treatment	General anesthesia	10 mg Midazolam, 10 mg famotidine	50 mg propofol	6–10 mg/kg propofol, 67% nitrous oxide, 33% oxygen	NA	2 h	NA	No complications
		1	5	11	BMD with dental treatment	General anesthesia	6 mg Diazepam, 2.5 mg famotidin	5% sevoflurane, 67% nitrous oxide, 33% oxygen	6–12 mg/kg propofol, 0.5–1.5% sevoflurane, 67% nitrous oxide, 33% oxygen	NA	2 h 20 min	NA	No complications
Bush and Dubowitz (49)	UK	1	6	NA	BMD with dental treatment	General anesthetic	Diazepam	Nitrous oxide, oxygen and halothane	NA	Calcium chloride, dantrolene	NA	NA	Cardiac arrest

All patients needed sedation or anesthesia before surgery or diagnostic procedures, had an American Society of Anesthesiologists (ASA) grade of I–III, and had no history of allergies. Most of the patients were male, whereas three were female and underwent radical mastectomy, cesarean section, and laparoscopic hysterectomy (25, 36, 43). The age ranged from 5 to 58 years. The sample sizes of the included studies varied between 1 and 29. The studies were conducted in Korea (*n* = 4), Iran (*n* = 2), China (*n* = 3), Germany (*n* = 2), America (*n* = 9), Turkey (*n* = 2), the UK (*n* = 3), the Netherlands (*n* = 2), Italy (*n* = 1), Japan (*n* = 5), France (*n* = 1), Brazil (*n* = 1), Belgium (*n* = 1), and India (*n* = 4) from 1995–2019. Some patients with DMD underwent general anesthesia for percutaneous nephrolithotomy, muscle biopsy, corrective orthopedic surgery, laparoscopic cholecystectomy, tumor excision, cholelithiasis, cholecystectomy, etc. Some other patients with DMD underwent local anesthesia for reduction internal fixation, upper extremity amputation, fistulectomy and intercostal nerve blocks. In addition, some patients with DMD underwent general anesthesia supplemented with regional anesthesia for laparoscopic cholecystectomy and traumatic cataract surgery. The duration of most anesthesia operations arranged from 30 min to 2 h.

### 3.2 Quality assessment (risk of bias assessment)

The quality of case report studies was evaluated using the JBI quality assessment tool. Thirty-five case report studies were included (4, 7, 22–54). The results of the quality evaluation revealed that 82.86% (29/35) of the studies described adverse events and unexpected events, 88.57% (31/35) of the studies clearly described patients' history and the researched intervention and/or treatment measures, 88.57% (31/35) of the studies clearly described patients' demographic characteristics. A total of 91.43% (32/35) of the studies clearly described the health status of the patients after the intervention, and 61.5% (8/13) of the studies described the implications of the study. A total of 85.71% (30/35) of the studies clearly presented the current clinical health problems of the patients. Eighty percent (28/35) of the studies clearly described the diagnosis, assessment method, and outcomes, indicating that the overall quality of case reports was high. The results of quality evaluation of the case reports are shown in Table 4.

**Table 4:** Quality evaluation results of case reports.

**References**	**Were patient's demographic characteristics clearly described?**	**Was the patients history described and presented as a timeline?**	**Was the current clinical condition of the patient on presentation clearly described?**	**Were diagnostic tests or assessment methods and the results clearly described?**	**Was the intervention(s) or treatment procedure(s) clearly described?**	**Was the post-intervention clinical condition clearly described?**	**Were adverse events or unanticipated events identified and described?**	**Does the case report provide takeway lessons?**
Kim and Chun (22)	Yes	No	Yes	Yes	Yes	Yes	Yes	Yes
Parish and Farzin (23)	Yes	Yes	Yes	Yes	Yes	Yes	Yes	Yes
Jung et al. (24)	No	No	Yes	Yes	Yes	Yes	No	Yes
Zhou et al. (25)	Yes	Yes	No	Yes	Yes	Yes	Yes	Yes
Bang et al. (26)	Yes	Yes	Yes	Yes	Yes	Yes	Yes	No
Frankowski et al. (4)	Yes	Yes	No	Yes	Yes	No	Yes	Yes
Büget et al. (27)	Yes	Yes	Yes	Yes	Yes	Yes	Yes	Yes
de Boer et al. (12)	Yes	Yes	Yes	Yes	Yes	Yes	Yes	Yes
Wefki Abdelgawwad Shousha et al. (13)	Yes	Yes	Yes	Yes	Yes	Yes	Yes	Yes
Obata et al. (28)	Yes	Yes	Yes	Yes	Yes	Yes	Yes	Yes
Iwata et al. (29)	No	No	Yes	Yes	Yes	Yes	No	Yes
Richa et al. (30)	Yes	Yes	Yes	Yes	Yes	Yes	Yes	Yes
Shimauchi et al. (14)	Yes	Yes	Yes	Yes	Yes	Yes	Yes	Yes
Saldanha et al. (31)	No	No	Yes	No	Yes	Yes	Yes	Yes
Kocabas et al. (32)	Yes	Yes	Yes	Yes	Yes	Yes	No	Yes
Smelt (15)	Yes	Yes	Yes	No	Yes	Yes	Yes	Yes
Irwin and Henderson (16)	Yes	Yes	Yes	Yes	Yes	Yes	Yes	Yes
Rajmala et al. (33)	Yes	Yes	Yes	No	Yes	Yes	Yes	Yes
Jain (34)	Yes	Yes	Yes	Yes	Yes	Yes	Yes	Yes
Vandepitte et al. (35)	Yes	Yes	Yes	Yes	Yes	Yes	No	Yes
Molyneux (36)	Yes	Yes	Yes	Yes	Yes	Yes	Yes	Yes
Van Obbergh et al. (37)	Yes	Yes	Yes	Yes	Yes	Yes	Yes	Yes
Peng and Wei (38)	Yes	Yes	Yes	Yes	Yes	Yes	Yes	Yes
Horikoshi et al. (39)	Yes	Yes	No	Yes	Yes	Yes	Yes	Yes
Sethna et al. (8)	No	Yes	Yes	Yes	Yes	Yes	Yes	Yes
Chalkiadis and Branch (41)	Yes	Yes	Yes	Yes	Yes	Yes	Yes	Yes
Shafy et al. (42)	Yes	Yes	Yes	No	Yes	Yes	Yes	Yes
Rathi et al. (43)	Yes	Yes	No	Yes	Yes	Yes	Yes	Yes
Kulshrestha et al. (45)	Yes	Yes	Yes	No	Yes	No	Yes	Yes
Raman et al. (46)	Yes	Yes	Yes	No	Yes	Yes	No	Yes
Rozmiarek et al. (7)	Yes	Yes	Yes	Yes	Yes	No	No	Yes
Wang and Stanley (47)	Yes	Yes	Yes	Yes	Yes	Yes	Yes	Yes
Kim et al. (48)	Yes	Yes	Yes	Yes	Yes	Yes	Yes	Yes
Bush and Dubowitz (49)	Yes	Yes	No	Yes	Yes	Yes	Yes	Yes
Kawaai et al. (44)	Yes	Yes	Yes	No	Yes	Yes	Yes	Yes

A methodological appraisal of the selected non-randomized studies using the MINORS tool is presented in Table 5 (50–54). The assessment scores ranged from 11 to 13, with a maximum global score of 16. Three studies clearly stated the aim of the investigation, reported the prospective collection of data, and properly described the main outcomes. Additionally, no loss of treated subjects during the follow-up period was reported. However, limitations were found in the description of the inclusion of consecutive patients and in the prospective calculation of the study size (50–54). Thus, three studies were classified as “moderate quality” (51, 53, 54) and two studies as “high quality” (50, 52).

**Table 5:** Quality evaluation results of non-randomized controlled study.

**References**	**A clearlystated aim**	**Inclusion of consecutive patients**	**Prospective collection of data**	**Endpoints appropriate to the aim of study**	**Unbiased assessment of the study endpoint**	**Follow-up period appropriate to the aim of the study**	**Loss to follow up <5%**	**Prospective calculation of the study size**	**An adequate control group**	**Contemporary groups**	**Baseline equivalence of groups**	**Adequate statistical analyses**
Ihmsen et al. (50)	2	1	1	2	1	0	1	0	1	2	1	1
Wick et al. (51)	2	1	1	2	1	0	1	0	1	1	1	1
Muenster et al. (52)	2	1	1	2	1	0	1	0	1	2	1	1
Ririe et al. (53)	2	1	1	2	1	0	1	0	1	1	1	1
Kako et al. (54)	2	1	1	1	1	0	1	0	1	1	1	1

The total score of MINORS scale was 16 points. 0: the literature has not been reported, 1: the literature has been reported but the information was insufficient, 2: the literature has been reported and sufficient information has been provided, and a total score of ≥13 was classified as high quality literature.

### 3.3 Preoperative evaluation

The cardiac status needs to be carefully considered in the preoperative evaluation of DMD patients. Most of the included studies performed electrocardiography or pulmonary auscultation before surgery and reported the results. The American Society of Anesthesiologists' physical status of the patients was reported to be I–III (13, 14, 31, 51). The preoperative evaluation should focus on the end-organ involvement of DMD, its evaluation, and the development of an anesthetic drug plan based on these findings. In addition to cardiac involvement, as noted above, respiratory involvement is universally present in patients with DMD. For a full discussion regarding the respiratory concerns of patients with DMD, the reader is referred to the review in the journal written by the pulmonologists who participated in the development of the consensus statement from the American College of Chest Physicians (55).

### 3.4 Pharmacological interventions

Tables 1, 2 present the drug management strategies used for DMD patients during the induction, maintenance, and recovery periods from anesthesia. Five non-randomized controlled trials compared the DMD group with the control group. Wick et al. (51) determined the onset time and complete spontaneous recovery from neuromuscular blockade after the administration of a standard dose of 0.6 mg/kg rocuronium in patients with advanced DMD compared with controls. Ihmsen et al. (50) compared children with DMD with normal patients to investigate the effects of mivacurium on neuromuscular blockade. Tino Muenster et al. investigated the onset time, peak effect and complete spontaneous recovery from neuromuscular blockade after the administration of a single dose of 0.3 mg/kg rocuronium in DMD patients and compared the data with those of controls (52). Ririe et al. (53) used vecuronium to characterize the neuromuscular blockade of patients with DMD and the response to that of the controls. In addition, Kako et al. (54) evaluated a combination of ketamine with two different doses of dexmedetomidine for sedation during muscle biopsy in patients with DMD.

#### 3.4.1 Main mode of anesthesia

Twenty-six of the included studies involved general anesthesia (4, 12–16, 22–24, 28, 30–32, 37, 39–41, 44, 47–54). Standard intraoperative monitoring, including electrocardiography, automatic blood pressure, and pulse oximetry, was used in the studies. General anesthesia was mostly induced with propofol, fentanyl, pentothal sodium, and midazolam (22–26, 50–52). Rocuronium was administered as a muscle relaxant (14, 15, 24, 32, 39, 48). Anesthesia was mostly maintained with propofol and remifentanil with an oxygen–air mixture (13, 14, 22–25, 28–31). Moreover, a few studies used inhalation induction for general anesthesia with sevoflurane and nitrous oxide (28, 40, 41).

Six studies included regional anesthesia (26, 27, 35, 36, 42, 43). These studies reported local nerve blockade with lidocaine, ropivacaine and other drugs. These studies suggested that general anesthesia or central neuraxial blockade in patients with severe DMD is an unsafe approach to anesthesia because of hemodynamic instability and respiratory depression. Peripheral nerve block is the best way to reduce the risk of critical complications and is a safe and feasible approach to anesthesia in patients with severe DMD.

Four of the included studies involved general anesthesia supplemented with regional anesthesia (29, 33, 34, 38). Anesthesia was induced and maintained with propofol, remifentanil, and fentanyl; local nerve block with ropivacaine and lidocaine was performed.

Moreover, four of the included studies involved the use of procedural sedation (7, 45, 46, 54). Procedural sedation was induced and maintained with dexmedetomidine, ketamine and midazolam (7, 45, 46, 54).

#### 3.4.2 Anesthesia premedication

Nineteen studies involved the use of anesthesia drugs as a premedication (4, 12, 13, 16, 22, 23, 30, 31, 42, 44–47, 49–54). The drugs used included midazolam, acetaminophen, morphine, ondansetron, and hydroxyzine. Kim and Chun (22) reported that 0.2 mg of glycopyrrolate was injected intramuscularly for anesthesia premedication. Parish and Farzin (23) reported that a total of 200 mg of hydrocortisone was injected as the stress dose. Some studies have used midazolam as an anesthesia premedication at doses ranging from 1 to 5 mg (4, 50–54).

#### 3.4.3 Anesthesia induction

Twenty-three studies involved the use of anesthesia induction drugs (4, 13–16, 22–25, 28–33, 37–39, 41, 44, 47–49). The drugs used included midazolam, pentothal sodium, rocuronium bromide, sodium thiopental, propofol, and fentanyl. In patients in whom a muscle relaxant is used, monitoring of muscle relaxation was performed via acceleromyography. The agents used for anesthetic induction should be based on the patient's comorbid cardiac condition. Although the effect of etomidate on adrenal function has led to a re-evaluation of its use during endotracheal intubation in critically ill ICU patients, it may still be an appropriate choice for anesthetic induction in patients with diminished myocardial function (56). The depolarizing agent succinylcholine is absolutely contraindicated and should not even be drawn into a syringe. Rocuronium bromide, with its usually short onset time, could be a suitable alternative to succinylcholine in DMD patients when the clinical conditions require rapid muscle relaxation for airway protection. When motor-evoked potentials are used to monitor spinal cord function, a single dose of a non-depolarizing NMBA can be used to facilitate endotracheal intubation. However, in patients with myopathic conditions such as DMD, the duration of blockade is prolonged (57).

**Figure 2:**
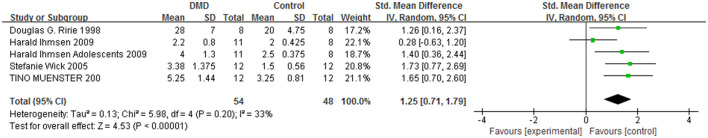
Meta-analysis of the onset time (min).

**Figure 3:**
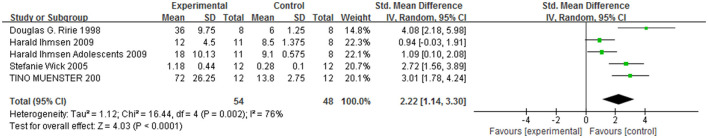
Meta-analysis of the recovery time (min).

Moreover, five non-randomized controlled trials, including a total of 155 patients, compared the DMD group with the control group to determine the onset time and recovery time after the administration of a standard dose of NMBAs in patients (50–54). Compared with the control group, the sensitivity of patients with DMD to NMBAs may result in a prolonged onset time [MD = −0.96, 95% CI (0.71, 2.60), *I*^2^ = 33%, *P* < 0.0001; Figure 2] and recovery time [MD = 2.22, 95% CI (1.14, 3.30), *I*^2^ = 76%, *P* < 0.0001; Figure 3] from anesthesia. Therefore, the anesthetic management of these patients is challenging and may cause serious problems for anesthesiologists. A sensitivity analysis of each comparison revealed no robust changes in significance.

Similarly, Jung et al. (24) documented that the responsiveness of DMD patients administered a standard dosage of non-depolarizing NMBAs differs from that of normal patients. The delayed onset of blockade in DMD patients following the administration of standard-dose rocuronium and prolonged recovery from rocuronium-induced blockade necessitate the need for a careful assessment of neuromuscular function. If rocuronium is administered to patients with DMD, an quantitative assessment of complete neuromuscular recovery, such as acceleromyography, is mandatory.

#### 3.4.4 Anesthesia maintenance

Thirty-two of the included studies involved anesthesia maintenance (4, 7, 12–16, 22–25, 28–34, 37, 39–41, 44–47, 49–54). Intravenous anesthetics such as propofol, fentanyl, remifentanil, ketamine, dexmedetomidine, and rocuronium bromide are reasonable alternatives and are commonly used at variable doses (Table 1). In addition, some studies used inhalation anesthesia with nitrous oxide, oxygen, sevoflurane, isoflurane and halothane to maintain anesthesia (40, 41, 44).

Richa et al. (30) recommended remifentanil for children with DMD. They reported that the combination of propofol and remifentanil infusions with nitrous oxide in oxygen was successful for patients with DMD undergoing spinal surgery. Exaggerated reactions to drugs were not observed. The patient's intraoperative blood pressure and heart rate were stable, and the wake-up test was successful. Alternatively, endotracheal intubation can be accomplished with a combination of propofol and remifentanil to avoid the need for a neuromuscular blocking agent (41).

Moreover, the multidisciplinary panel suggested the use of total intravenous anesthesia (TIVA) to induce and maintain general anesthesia (e.g., propofol and short-acting opioids) (55). Maintenance anesthesia during surgery for scoliosis generally includes TIVA not only due to the abovementioned concerns of rhabdomyolysis related to volatile anesthetic agents but also to facilitate neurophysiological monitoring using motor and somatosensory evoked potentials. Despite the popularity and clinical experience of the use of propofol for TIVA in these patients, recent concerns have been expressed regarding the effect of propofol on mitochondrial oxidative function (10). These concerns have been raised because rhabdomyolysis, which is thought to be secondary to the disruption of mitochondrial fatty acid oxidation, can occur with prolonged propofol infusion in the pediatric ICU setting, and a defect in mitochondrial oxidative capacity is known to occur in patients with muscular dystrophies (58–61). Despite such concerns, TIVA with propofol and a synthetic opioid remains the most commonly chosen anesthetic regimen (11). However, dexmedetomidine may be added to decrease the propofol dose (62, 63). Kako et al. (54) reported that the use of dexmedetomidine (0.5 μg/kg) and ketamine (1 mg/kg) as loading doses followed by continuous infusion of 0.5 kg/kg/h dexmedetomidine achieved the appropriate sedation level with a shorter total recovery time than the higher-dose dexmedetomidine regimen. Therefore, the combination of dexmedetomidine and ketamine is safe and effective for moderately painful procedures with limited respiratory and cardiovascular effects on high-risk patients.

#### 3.4.5 Adverse events of anesthesia

The sensitivity of patients with DMD to sedative, anesthetic and neuromuscular blocking agents may result in intraoperative and early postoperative cardiovascular and respiratory complications, as well as prolonged recovery from anesthesia. When sedation/anesthetic was excessive, sedation/anesthesia reversal was particularly necessary. Sixteen of the included studies involved the use of anesthesia reversal (12–16, 22, 24, 28, 33, 34, 38–41, 47, 49). Sugammadex, neostigmine and atropine were administered at different doses to reverse cisatracurium besylate-induced neuromuscular blockade (Table 1).

These studies described the efficacy of sugammadex for reversing a prolonged blockade in this setting, but no adverse events were observed (12–14, 22). Jung et al. (24) reported the use of 4 mg of pyridostigmine and 0.16 mg of glycopyrrolate to reverse deep NMB in a child with DMD. Rajmala et al. (33) used 0.05 mg/kg neostigmine and 0.02 mg/kg atropine after the appearance of respiratory efforts, and the postoperative course was uneventful. Similarly, Peng and Wei (38) used 0.2 mg of neostigmine and 0.1 mg of atropine to reverse deep NMB. Treatment with inotropic agents such as milrinone or dobutamine may be necessary to support myocardial function. Close monitoring of cardiac rhythm should be standard, and rhythm abnormalities should be promptly treated (57). Pyridostigmine has been shown to be an effective reversal agent in patients with DMD (24).

Notably, many reports of fatal hyperkalemic cardiac arrest associated with the use of succinylcholine in patients with DMD have raised anesthetists' awareness of this potential complication (16, 28, 40, 41, 47, 49). Therefore, the anesthesia community now commonly accepts that this drug should be strictly avoided in patients with DMD (11). However, rhabdomyolysis may occur in the absence of succinylcholine intraoperatively and during postoperative cardiac arrest as a result of hyperkalemia in patients with DMD (16, 27, 28, 40, 41, 47, 49, 64). The eventual contribution of general anesthetic agents to the cause of the event cannot be ascertained because events occurred during IV and inhaled anesthetic exposure without succinylcholine (16, 28). Moreover, we identified seven cases of rhabdomyolysis and intraoperative cardiac arrest secondary to hyperkalemia during the use of the inhaled anesthetics isoflurane, halothane, and sevoflurane (16, 28, 40, 41, 47, 49). In these patients, a clear precipitant rhythm or event was difficult to discern. Resuscitations persisted in excess of 60 min, with full recoveries obtained in six patients. However, one patient was discharged home with no subjective changes in cerebral function. However, he was paraplegic (sensory level T) (41). Dantrolene is often used empirically after documented concomitant metabolic and respiratory acidosis, with or without modest temperature increases. These cases suggest a predisposition to rhabdomyolysis upon exposure to volatile anesthetics, regardless of surgical stress. The disease is not known to be associated with MH; the components of effective resuscitation are difficult to discern, but a reduction in serum potassium levels is crucial (28, 40, 41, 47, 49).

#### 3.4.6 Postoperative pain control

Appropriate analgesics should be encouraged to provide postoperative analgesia without affecting the patient's normal respiratory function. As in other patients, the analgesic drugs of choice for patients with DMD are opioids. Depending on the duration of the surgical intervention, the choice of opioid should be based on the pharmacological effect and pharmacokinetics. We have administered nearly all clinically used opioids in our series (Tables 1, 2). Six of the included studies investigated postoperative pain control (14, 16, 25, 26, 28–32, 35, 37, 39, 42, 43, 48, 54). Postoperative interventions, such as paracetamol, PCA, morphine pumps, pethidine, nalbuphine, dipirone, flurbiprofen axetil, diclofenac, sufentanil, ketorolac and atropine, were administered at different doses to provide postoperative analgesia (Table 1).

Given the severity of the surgical procedure, several options exist for the provision of postoperative analgesia. In patients undergoing spinal fusion surgery, neuraxial techniques have been used to achieve analgesia through the intermittent or continuous infusion of opioids and/or local anesthetics via epidural catheters, with minimal respiratory side effects (65).

In addition, given their effects on the central control of ventilation and cough effort, options that limit the use of opioids, including adjunct agents or regional anesthesia, should be considered. Preliminary data from the adult population have shown the potential role of the preoperative administration of pregabalin or gabapentin (66). Additionally, postoperative administration of the a2-adrenergic agonist dexmedetomidine and intravenous acetaminophen may play a role. Moreover, caution has been suggested with the use of non-steroidal anti-inflammatory agents given their anecdotal and temporal association with rhabdomyolysis (67, 68).

## 4 Discussion

Patients with DMD are uniquely vulnerable to the adverse physiological effects of general anesthesia and procedural sedation (55). Tachycardia, ventricular fibrillation and cardiac arrest have been reported during the induction of anesthesia (40, 41, 69–71). In almost all patients assessed in these case reports, DMD was not suspected until a further investigation was prompted by the occurrence of cardiac manifestations. In addition, ventricular fibrillation or cardiac arrest has also been described in patients who are known to have DMD following a return of consciousness while the patient is still in the recovery room (72). Therefore, patients with DMD should receive a detailed preoperative assessment, thoughtful disease-specific intraoperative management and aggressive postoperative monitoring if they are to avoid anesthesia- and surgery-related morbidity and mortality. Moreover, all children presenting for the administration of general anesthesia or sedation should be screened for motor milestones. The inability to walk at an age >18 months or other signs of motor loss or elevated levels of CPK should prompt a suspicion of subclinical myopathy and should warrant a neurological evaluation and genetic testing before elective surgery. Most cases of DMD are detected via genetic testing (55). In addition, timing disease-related major surgical procedures, such as scoliosis surgery, early in a child's life prior to the onset of significant myocardial dysfunction is recommended to minimize the cardiovascular risk. Finally, surgery for DMD patients should be performed in a hospital equipped to address the unique issues faced by patients with neuromuscular disorders (57).

In addition, because postoperative pulmonary complications might be one of the causes of postoperative complications in DMD patients, before general anesthesia or procedural sedation, the following lung function parameters should be measured to assess the patients' risk of respiratory complications and the need for perioperative and postoperative assisted ventilation or cough. The application of non-invasive ventilation modalities in the preoperative and postoperative setting to limit pulmonary postoperative complications using personalized non-invasive respiratory support is important (55). Options for respiratory support include manual ventilation using a flow-inflated manual resuscitation bag (standard “anesthesia bag”) with a full face or nasal mask interface and mechanical support using a conventional or non-invasive positive pressure ventilator via a full face or nasal mask.

Furthermore, these included case studies also revealed that general anesthesia and central neuraxial blockade in patients with severe DMD are unsafe approaches to anesthesia. Peripheral nerve blocks are the best way to reduce the risk of critical complications and are a safe and feasible approach to anesthesia in patients with severe DMD (26, 27, 42, 43). Notably, the general anesthetics that resulted in cardiac complications at induction were succinylcholine and volatile anesthetics. Therefore, the anesthesia community now commonly accepts that anesthetic machines free of volatile agents (including a new disposable breathing circuit) should be used and that succinylcholine is avoided. Monitoring should include a temperature probe, an ECG, and a nerve stimulator (73).

In addition, although general anesthesia may be required for specific procedures, moderately painful procedures such as bone marrow aspiration and biopsy can be performed with procedural sedation and the maintenance of spontaneous ventilation. Given these issues, a need remains for a better agent or agents for procedural sedation. The current evidence suggests the use of a total intravenous anesthesia (TIVA) technique to induce and maintain general anesthesia (e.g., propofol and short-acting opioids) and is advised rather than the use of depolarizing muscle relaxants (48). The authors reported their experience with a combination of ketamine and dexmedetomidine for sedation during bone aspiration and biopsy in an adolescent with DMD (7), which revealed that the application of dexmedetomidine in patients with DMD has the potential to be a promising treatment option in the future.

Moreover, the sensitivity of patients with DMD to NMBAs may result in prolonged onset and recovery times from anesthesia. Muenster et al. speculated that one reason for the prolonged duration of NMB in these patients could be the known degradation of muscle fibers and their replacement by fatty and fibrous tissue with the progression of the disorder. These structural changes are obviously accompanied by a decrease in the total number of neuromuscular junctions and receptors. Consistently, in an experimental study in mdx mice, accelerated degradation of adult nicotinic acetylcholine receptors was observed (74). Such a situation with a reduced number of receptors strongly influences the dose–response relationship of administered non-depolarizing NMBA. Therefore, the wide interpatient variability in the recovery time after the administration of a reduced dose does not allow an estimation of the time needed for complete recovery in a single patient (52). In particular, regarding the prolonged onset time, special attention must be paid to the effect of the relaxant agent used. The use of muscle relaxants is a major concern when performing anesthesia in DMD patients (52). Several prospective investigations have shown that nearly all commonly used non-depolarizing NMBAs can be used in patients with DMD (11). This situation is especially true for rocuronium and mivacurium (11, 52). These reports also revealed that the response to non-depolarizing NMBAs is altered in patients with DMD. The most striking difference is the delayed onset of blockade in DMD patients compared with normal patients. This effect should be considered in situations where rapid airway protection is necessary. Another significant difference is the prolonged duration of recovery from NMB in DMD following standard doses of non-depolarizing NMBAs. Notably, depending on the time of reversal, the duration of residual block after rocuronium may exceed the duration of antagonism by the reversal agent. Therefore, using reversal agents in this situation involves the risk of possible “recurarization” (51). Therefore, even after the administration of a reversal agent, monitoring of muscle strength in the recovery room either quantitatively or clinically should be performed (51). Furthermore, these effects depend on the stage of the disease, with more pronounced effects observed with ongoing progression. This altered response to non-depolarizing NMBAs in patients with DMD makes a quantitative assessment of complete neuromuscular recovery, such as acceleromyography, necessary (11).

In addition, the existing evidence implicates calcium dysregulation as an underlying crucial event in the pathophysiology of DMD (73). In malignant hyperthermia, defective influx and efflux of Ca from the sarcoplasmic reticulum has been observed in mouse models. Since a malignant hyperthermia-like syndrome may occur in DMD patients during anesthesia, maneuvers capable of reducing Ca influx into cells have beneficial effects on these patients DMD; thus, the possibility that a reduction in Ca influx from the sarcoplasmic reticulum by a Ca antagonist, such as dantrolene, may result in additional benefits for patients with DMD (74).

Several limitations of the study should be acknowledged. The strengths of this systematic review include the broad and complete search strategy, the publication of a protocol a priori, and the validated methodology used to assess the included studies, e.g., Cochrane's “Risk of Bias” 2.0 tool and ROBINS-I. We adhered to the protocol to minimize intellectual bias in conducting and reporting the findings. Two authors independently screened studies for inclusion and performed the risk-of-bias assessment. Potential limitations include our broad approach, i.e., for example, we included all studies regardless of the type of drugs, which may have contributed to the high degree of clinical heterogeneity of the included studies. Furthermore, our choice of the definition of outcomes could be discussed. We chose analgesia, sedation and mortality at discharge as the primary outcomes. Unfortunately, a possibility of poorly documented minor complications or minor adverse events caused by anesthetic medication always exists. Finally, we did not explore the significance of a diagnosis of respiratory or cardiac involvement in the prediction of perioperative complications in DMD patients, and we were not able to perform a retrospective evaluation. No cases of patients requiring postoperative ventilatory support were documented, regardless of whether NMBAs had been used. Follow-up examinations after adverse reactions to general anesthesia are often incomplete, and some patients receive the same type of anesthesia again (75). Whether the negative responses in DMD patients originate during multiple exposures to anesthesia or sedation is unknown, and additional high-quality research is needed to provide more comprehensive information.

Finally, the primary difference between DMD and BMD is the quantity of dystrophin present in skeletal and cardiac muscle. In patients with DMD, dystrophin is almost always absent, whereas partially functional dystrophin is present in patients with BMD and results in a milder form of the disorder and longer survival, which was consistent with the data in Tables 2, 3. However, 2 patients with BMD who were very young were described (23, 49). In addition, compared with patients with BMD, patients with DMD had more comorbid conditions and higher rates of cardiomyopathy and severe restrictive lung disease. However, patients with BMD are present with serious postoperative adverse reactions (49). Postoperatively, DMD and BMD patients must be monitored until cardiorespiratory function returns to the baseline. The current case reports are insufficient for generating definitive conclusions regarding the significant differences between patients with BMD and DMD. Overall, the anesthesia technique must be customized and adjusted for each patient.

## 5 Conclusions

The results of the included studies confirmed that patients with DMD are more sensitive to NMBAs, which may result in a delayed onset time and prolonged recovery time from anesthesia, and these effects depend on the stage of the disease, with more pronounced effects observed with ongoing progression. Precautions for DMD patients should include quantitative neuromuscular and electrocardiographic monitoring and rapid airway protection throughout anesthesia. The strict avoidance of succinylcholine and volatile anesthetics during anesthesia in patients with DMD can prevent known anesthetic hazards such as rhabdomyolysis or hypercalcemia. Compared with general anesthesia, regional anesthesia can be a relatively safe option (if the surgical site is appropriate for the technique). Dantrolene should be available in the theater and be readily used if events consistent with a malignant hyperpyrexial response to anesthesia occur. However, further prospective clinical trials are needed to determine the most effective interventions for patients with DMD.

## Data Availability

The original contributions presented in the study are included in the article/supplementary material, further inquiries can be directed to the corresponding author.
